# NPHR, FPR, and PLT as predictors of ulcerative colitis severity: a single-center retrospective study in China

**DOI:** 10.3389/fmed.2026.1814784

**Published:** 2026-05-04

**Authors:** Shaolei Yang, Dandan Liang, Yufen Qin, Fengxian Dai, Guangxi Zhou, Fengqin Zhu

**Affiliations:** 1 Clinical Medical College of Jining Medical University, Jining Medical University, Jining, Shandong, China; 2 Department of Gastroenterology, Affiliated Hospital of Jining Medical University, Jining Medical University, Jining, Shandong, China

**Keywords:** disease activity, fibrinogen-to-prealbumin ratio, neutrophil-to-hemoglobin ratio, platelets, ulcerative colitis

## Abstract

**Background:**

Ulcerative colitis (UC) is a chronic inflammatory bowel disease, and precise evaluation of disease activity is essential for effective management. This study aimed to assess the value of the peripheral-blood neutrophil percentage-to-hemoglobin ratio (NPHR), fibrinogen-to-prealbumin ratio (FPR), and platelet count (PLT) in identifying moderate-to-severe UC and to develop a predictive model for clinical risk stratification.

**Methods:**

Data from 277 hospitalized UC patients, consecutively admitted to the Department of Gastroenterology at the Affiliated Hospital of Jining Medical University between May 2023 and October 2025, were retrospectively analyzed. Disease activity was classified based on the modified Mayo score into low-activity (remission or mild) and moderate-to-severe groups. Independent predictors were identified using multivariable logistic regression and incorporated into a nomogram. Discrimination and calibration were assessed via receiver operating characteristic (ROC) curves and calibration curves, respectively. Clinical utility was evaluated through decision curve analysis (DCA) and the clinical impact curve (CIC).

**Results:**

NPHR, FPR, and PLT were independent risk factors for moderate-to-severe UC (*p* < 0.05). The areas under the ROC curves for NPHR, FPR, and PLT were 0.823 (95% CI, 0.773–0.873), 0.788 (95% CI, 0.733–0.842), and 0.746 (95% CI, 0.687–0.805), respectively. The calibration curve demonstrated strong agreement with the ideal line. DCA revealed a favorable net benefit within a threshold probability range of 0.16–1.0, while CIC confirmed the model’s high clinical utility in the range of 0.30–0.90.

**Conclusion:**

NPHR, FPR, and PLT are independent predictors of moderate-to-severe UC. The combination of these markers enhances predictive accuracy and provides a valuable tool for disease activity assessment.

## 1 Introduction

Ulcerative colitis (UC) represents a prolonged, recurrent inflammatory state characterized by the predominant involvement of the colorectal mucosa. As the disease progresses, patients may develop a wide range of complications that significantly impair quality of life. Although the precise etiology remains unclear, UC is generally believed to emerge from a combination of genetic predisposition, immune dysregulation, microbiome shifts, and environmental exposures (1). Recently, the global prevalence of UC has increased substantially, making it a growing public health concern (2). Currently, colonoscopy with histopathological examination serves as the benchmark for assessing disease activity. Nevertheless, its invasiveness, procedure-related risks, high cost, and limited patient acceptability hinder its repeated use and pose challenges for long-term monitoring in routine clinical practice. Consequently, there is an increasing need for biomarkers that enable noninvasive, dynamic monitoring of disease activity, assessment of therapeutic responses, and prediction of relapse. Noninvasive serologic and fecal biomarkers, due to their accessibility and ease of measurement, have become a focal point in UC research.

Clinically utilized biomarkers can be broadly categorized into fecal and serologic types. Fecal markers such as fecal calprotectin (FC) and lactoferrin reflect intestinal inflammation directly (3, 4). However, their diagnostic performance can be influenced by factors such as sampling intervals, medication use, and gastrointestinal bleeding. Additionally, stool collection can be cumbersome, and patient adherence is often suboptimal. In contrast, commonly used serologic markers, including the erythrocyte sedimentation rate (ESR), C-reactive protein (CRP), and procalcitonin, are readily accessible but often lack adequate sensitivity and specificity for evaluating UC inflammatory activity (5). These limitations highlight the need for a novel biomarker that is rapid, stable, low-cost, and highly acceptable to patients, thereby enhancing disease assessment, treatment decision-making, and prognostic monitoring.

During active UC, neutrophils (NEU) infiltrate the affected mucosa (6), while reduced hemoglobin (Hb) may reflect increased systemic consumption. The NEU percentage-to-Hb ratio (NPHR), a composite index that reflects both inflammatory burden and nutritional status, may have significant potential for assessing disease activity and severity in UC. To date, no study has specifically explored the association between NPHR and UC disease activity. Additionally, fibrinogen (FIB), prealbumin (PA), and platelet count (PLT) are involved in inflammatory responses and oxidative stress, and may serve as clinically relevant biomarkers (7–9). However, evidence on the FIB-to-PA ratio (FPR) and PLT in UC remains sparse. Therefore, this study compared NPHR, FPR, and PLT levels among UC patients with varying activity levels, examined their relationships with disease activity, and evaluated their clinical relevance in assessing disease severity to inform clinical practice.

## 2 Materials and methods

### 2.1 Study design and patient population

This study included hospitalized UC patients who met the inclusion criteria and were consecutively recruited from the Department of Gastroenterology at the Affiliated Hospital of Jining Medical University, from May 2023 to October 2025. Patients were eligible if they met the following criteria: (1) UC diagnosis according to the 2023 Chinese clinical practice guideline on ulcerative colitis management (Xi’an) (10); (2) completion of a full colonoscopy, with all laboratory tests conducted within 3 days prior to endoscopic examination. Exclusion criteria were as follows: (1) infections involving the respiratory tract, urinary tract, skin, or soft tissues, or other systems; (2) comorbid hepatic, renal, cardiovascular, or cerebrovascular diseases; (3) malignancies or other autoimmune diseases; (4) pregnancy or lactation.

### 2.2 Data collection

Recorded demographic and clinical data included age, sex, height, weight, smoking and alcohol consumption, disease duration, colonoscopic findings, and laboratory results. Laboratory variables included complete blood count parameters and additional indices, such as CRP, PA, FIB, and D-dimer. Disease activity was evaluated with the modified Mayo score (0–12) (10), which includes stool frequency, rectal bleeding, endoscopic subscore, and physician’s global assessment, each scored 0–3. Disease activity was categorized as remission (0–2), mild (3–5), moderate (6–10), or severe (11–12). Participants were divided into a low-activity group (*n* = 100; remission *n* = 26; mild activity *n* = 74) and a moderate-to-severe activity group (*n* = 177; moderate activity *n* = 133; severe activity *n* = 44).

### 2.3 Statistical analysis

Statistical analyses were performed with SPSS 26.0 and R 4.3.1. Continuous variables were tested for normality, with non-normally distributed data presented as median (interquartile range, IQR) and compared between groups using the Mann–Whitney *U* test. Categorical variables were presented as *n* (%) and analyzed with the *χ*^2^ test. Spearman correlation analysis evaluated associations between study indices and the Mayo endoscopic score (MES) (11), Ulcerative Colitis Endoscopic Index of Severity (UCEIS) (12), modified Mayo score, and DUBLIN score (13). Prior to multivariable logistic regression, collinearity diagnostics were performed using tolerance and variance inflation factor (VIF) to assess potential multicollinearity among candidate variables. Logistic regression was used to identify factors associated with moderate-to-severe disease activity and to develop a prediction model, which was visualized as a nomogram. Internal validation was performed using bootstrap resampling (1,000 iterations), and model goodness-of-fit was assessed using the Hosmer–Lemeshow test. Model performance and clinical utility were assessed by receiver operating characteristic (ROC) curves, calibration curves, decision curve analysis (DCA), and clinical impact curves (CIC). A two-sided *p*-value <0.05 was regarded as statistically significant.

## 3 Results

### 3.1 Clinical characteristics

A total of 277 UC patients were included in the study: 100 in the low-activity group and 177 in the moderate-to-severe activity group. No significant differences were observed between the groups in terms of age, sex, body mass index (BMI), smoking history, alcohol consumption, disease duration, or lymphocyte percentage (*p* > 0.05). Compared to the low-activity group, the moderate-to-severe activity group exhibited significantly higher bowel movement frequency, PLT, D-dimer, white blood cell count (WBC), NEU%, NPHR, FIB, CRP, FPR, and CRP/PA (*p* < 0.001), whereas albumin (ALB), Hb, and PA levels were significantly lower (*p* ≤ 0.001). Specifically, the median NPHR, FPR, and PLT values were 0.515 (0.453–0.631) vs. 0.408 (0.373–0.460), 0.018 (0.014–0.025) vs. 0.013 (0.010–0.015), and 282.000 (229.500–332.000) × 10^9^/L vs. 218.500 (190.500–260.000) × 10^9^/L, respectively (Table 1).

**Table 1:** Comparison of clinical data between the two patient groups.

Variables	Low-activity group (*n* = 100)	Moderate-to-severe group (*n* = 177)	*Z*/*t*/*χ^2^*	*p*
Age (years)	52.760 ± 14.567	50.051 ± 14.573	1.486	0.138
Sex			0.026	0.871
Male	64 (64.000)	115 (64.972)		
Female	36 (36.000)	62 (35.028)		
BMI (kg/m^2^)	23.475 ± 3.447	23.155 ± 3.582	0.714	0.476
Smoking	20 (20.000)	32 (18.079)	0.155	0.694
Alcohol consumption	19 (19.000)	30 (16.949)	0.185	0.667
Disease duration (months)	48.000 (10.000, 84.000)	36.000 (9.500, 66.000)	−1.033	0.302
Bowel movement frequency (times/day)	2.500 (1.500, 4.500)	4.500 (2.500, 6.500)	−4.346	<0.001
PLT (×10^9^/L)	218.500 (190.500, 260.000)	282.000 (229.500, 332.000)	−6.807	<0.001
ALB (g/L)	41.050 (38.825, 43.600)	38.000 (35.200, 41.350)	−4.765	<0.001
D-dimer (μg/mL)	0.365 (0.280, 0.470)	0.510 (0.365, 0.785)	−5.315	<0.001
WBC (×10^9^/L)	5.515 (4.623, 6.645)	6.840 (5.700, 9.270)	−6.175	<0.001
LYM (%)	28.524 ± 9.968	27.223 ± 11.012	0.977	0.330
NEU (%)	53.410 ± 10.377	64.253 ± 11.305	7.894	<0.001
HB (g/L)	130.000 (119.000, 142.750)	123.000 (107.000, 137.000)	−3.463	0.001
NPHR	0.408 (0.373, 0.460)	0.515 (0.453, 0.631)	−8.928	<0.001
FIB (g/L)	2.705 (2.400, 3.100)	3.400 (2.900, 4.005)	−7.576	<0.001
CRP (mg/L)	0.980 (0.500, 2.688)	5.360 (1.675, 18.370)	−7.452	<0.001
PA (mg/L)	223.267 ± 50.817	183.942 ± 52.767	6.037	<0.001
FPR	0.013 (0.010, 0.015)	0.018 (0.014, 0.025)	−7.950	<0.001
CRP/PA	0.004 (0.002, 0.014)	0.028 (0.008, 0.110)	−7.711	<0.001

BMI, body mass index; PLT, platelet count; ALB, albumin; WBC, white blood cell count; LYM, lymphocyte count; NEU, neutrophil count; HB, hemoglobin; NPHR, neutrophil percentage-to-hemoglobin ratio; FIB, fibrinogen; CRP, C-reactive protein; PA, prealbumin; FPR, fibrinogen-to-prealbumin ratio; CRP/PA, C-reactive protein-to-prealbumin ratio.

### 3.2 Identifying independent risk factors for moderate-to-severe UC

Moderate-to-severe UC activity (yes/no) was modeled as the dependent variable. Univariable logistic regression revealed significant associations between bowel movement frequency, PLT, albumin, D-dimer, WBC, NPHR, FPR, and CRP/PA with moderate-to-severe UC activity (*p* ≤ 0.001). Bowel movement frequency (OR = 1.186, 95% CI: 1.076–1.308, *p* = 0.001), PLT (OR = 1.013, 95% CI: 1.009–1.018, *p* < 0.001), D-dimer (OR = 6.546, 95% CI: 2.515–17.040, *p* < 0.001), WBC (OR = 1.504, 95% CI: 1.296–1.746, *p* < 0.001), NPHR (OR = 3.684, 95% CI: 2.505–5.419, *p* < 0.001), FPR (OR = 4.955, 95% CI: 2.800–8.770, *p* < 0.001), and CRP/PA (OR = 5.283, 95% CI: 2.193–12.728, *p* < 0.001) were identified as risk factors for moderate-to-severe UC activity, whereas ALB (OR = 0.930, 95% CI: 0.891–0.970, *p* = 0.001) was identified as a protective factor (Table 2).

**Table 2:** Univariate logistic regression analysis of factors influencing moderate-to-severe UC.

Variables	*B*	SE	Wald*χ*^2^	*p*	OR (95% CI)
Bowel movement frequency	0.171	0.050	11.706	0.001	1.186 (1.076–1.308)
PLT	0.013	0.002	32.747	<0.001	1.013 (1.009–1.018)
ALB	−0.073	0.022	11.211	0.001	0.930 (0.891–0.970)
D-dimer	1.879	0.488	14.816	<0.001	6.546 (2.515–17.040)
WBC	0.408	0.076	28.782	<0.001	1.504 (1.296–1.746)
NPHR^*^	1.304	0.197	43.864	<0.001	3.684 (2.505–5.419)
FPR^*^	1.600	0.291	30.191	<0.001	4.955 (2.800–8.770)
CRP/PA^*^	1.665	0.449	13.770	<0.001	5.283 (2.193–12.728)

PLT, platelet count; ALB, albumin; WBC, white blood cell count; NPHR, neutrophil percentage-to-hemoglobin ratio; FPR, fibrinogen-to-prealbumin ratio; CRP/PA, C-reactive protein-to-prealbumin ratio.

NPHR and CRP/PA were each multiplied by 10 before being included in the model, and FPR was multiplied by 100 before being included in the model.

*Indicates that NPHR and CRP/PA were each multiplied by 10 before being included in the model, and FPR was multiplied by 100 before being included in the model.

All variables had VIF values below 5 and tolerance values above 0.1, suggesting the absence of evident multicollinearity among the variables included in the model (Table 3). Variables with statistical significance in the univariate analysis were then entered into multivariable logistic regression. The results indicated that PLT (OR = 1.013, 95% CI: 1.009–1.018, *p* = 0.012), NPHR (OR = 3.684, 95% CI: 2.505–5.419, *p* < 0.001), and FPR (OR = 4.955, 95% CI: 2.800–8.770, *p* = 0.014) were independent predictors for moderate-to-severe UC activity (Table 4).

**Table 3:** Multicollinearity diagnostics for variables included in the multivariable model.

Variables	VIF	Tolerance
Bowel movement frequency	1.125	0.889
PLT	1.634	0.612
ALB	1.172	0.853
D-dimer	1.239	0.807
WBC	1.524	0.656
NPHR^*^	1.436	0.697
FPR^*^	2.652	0.377
CRP/PA^*^	2.460	0.407

PLT, platelet count; ALB, albumin; WBC, white blood cell count; NPHR, neutrophil percentage-to-hemoglobin ratio; FPR, fibrinogen-to-prealbumin ratio; CRP/PA, C-reactive protein-to-prealbumin ratio.

NPHR and CRP/PA were each multiplied by 10 before being included in the model, and FPR was multiplied by 100 before being included in the model.

*Indicates that NPHR and CRP/PA were each multiplied by 10 before being included in the model, and FPR was multiplied by 100 before being included in the model.

**Table 4:** Multivariate logistic regression analysis of factors influencing moderate-to-severe UC.

Variables	*B*	SE	Wald*χ*^2^	*p*	OR (95% CI)
Bowel movement frequency	0.063	0.063	1.005	0.316	1.066 (0.941–1.206)
PLT	0.007	0.003	6.365	0.012	1.007 (1.002–1.013)
ALB	0.001	0.029	0.002	0.967	1.001 (0.947–1.059)
D-dimer	0.637	0.524	1.480	0.224	1.891 (0.678–5.277)
WBC	0.147	0.100	2.134	0.144	1.158 (0.951–1.410)
NPHR^*^	0.973	0.218	19.842	<0.001	2.645 (1.724–4.059)
FPR^*^	0.872	0.354	6.071	0.014	2.392 (1.195–4.785)
CRP/PA^*^	0.017	0.282	0.004	0.952	1.017 (0.585–1.767)
Constant term	−8.806	1.973	19.925	<0.001	—

PLT, platelet count; ALB, albumin; WBC, white blood cell count; NPHR, neutrophil percentage-to-hemoglobin ratio; FPR, fibrinogen-to-prealbumin ratio; CRP/PA, C-reactive protein-to-prealbumin ratio.

NPHR and CRP/PA were each multiplied by 10 before being included in the model, and FPR was multiplied by 100 before being included in the model.

*Indicates that NPHR and CRP/PA were each multiplied by 10 before being included in the model, and FPR was multiplied by 100 before being included in the model.

### 3.3 Correlation analysis of NPHR, FPR, and PLT with activity scores

**Figure 1:**
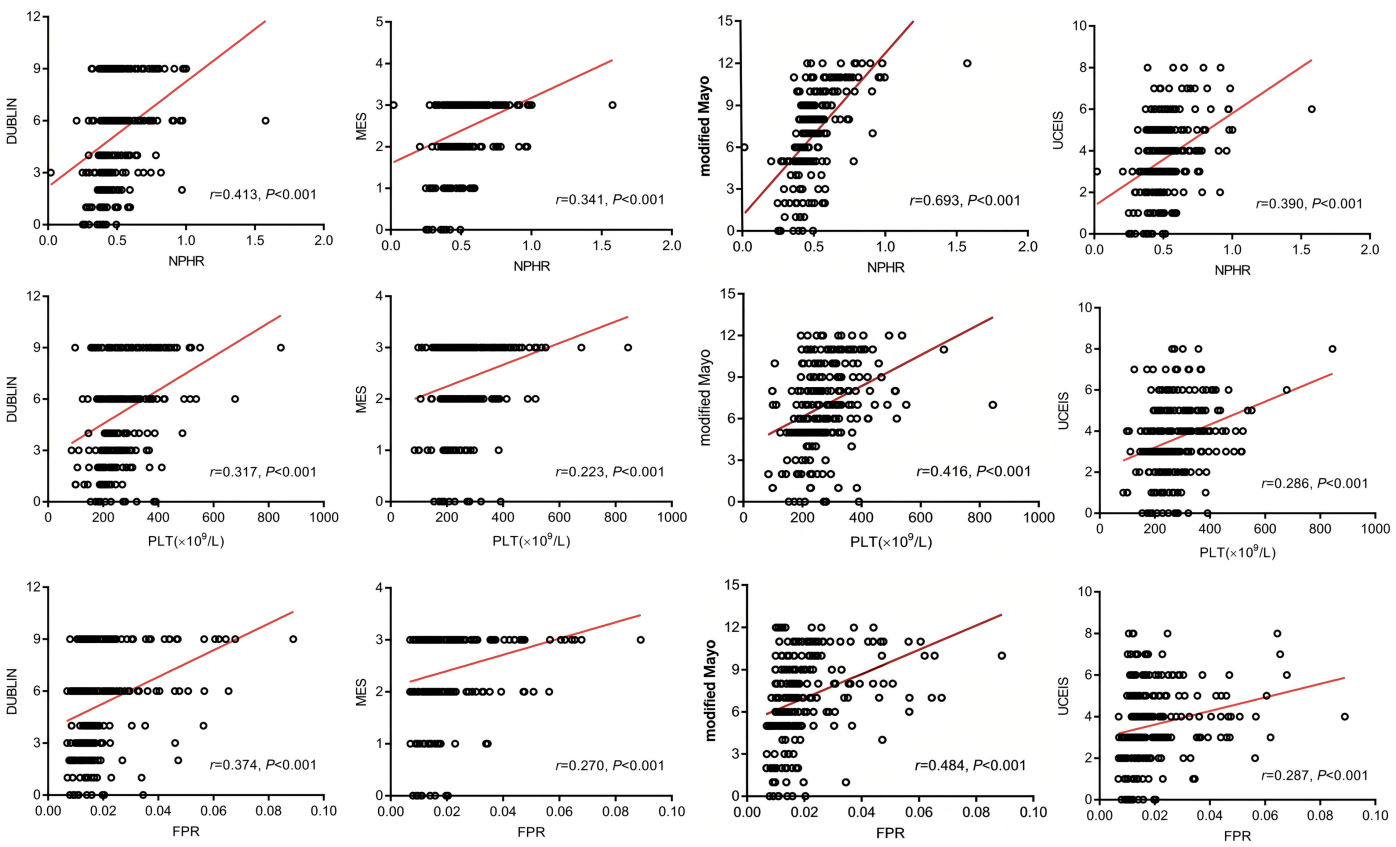
Correlation among various indicators. PLT, platelet count; NPHR, neutrophil percentage-to-hemoglobin ratio; FPR, fibrinogen-to-prealbumin ratio; MES, Mayo endoscopic score; UCEIS, ulcerative colitis endoscopic index of severity; DUBLIN, degree of ulcerative colitis burden of luminal inflammation.

Spearman correlation analysis demonstrated positive associations between NPHR, FPR, and PLT with MES, UCEIS, modified Mayo score, and the DUBLIN score (all *r* > 0; all *p* < 0.001). Among these markers, NPHR showed the strongest correlations with all disease activity indices, with correlation coefficients of 0.341, 0.390, 0.693, and 0.413 for MES, UCEIS, modified Mayo score, and the DUBLIN score, respectively. The corresponding correlation coefficients were 0.223, 0.286, 0.416, and 0.317 for PLT, and 0.270, 0.287, 0.484, and 0.374 for FPR (Table 5 and Figure 1).

**Table 5:** Correlation analysis among indicators.

Variables	PLT		NPHR		FPR	
	*r*	*p*	*r*	*p*	*r*	*p*
MES	0.223	<0.001	0.341	<0.001	0.270	<0.001
UCEIS	0.286	<0.001	0.390	<0.001	0.287	<0.001
Modified Mayo	0.416	<0.001	0.693	<0.001	0.484	<0.001
DUBLIN	0.317	<0.001	0.413	<0.001	0.374	<0.001

PLT, platelet count; NPHR, neutrophil percentage-to-hemoglobin ratio; FPR, fibrinogen-to-prealbumin ratio; MES, Mayo endoscopic score; UCEIS, ulcerative colitis endoscopic index of severity; DUBLIN, degree of ulcerative colitis burden of luminal inflammation.

### 3.4 Model development and validation

**Figure 2:**
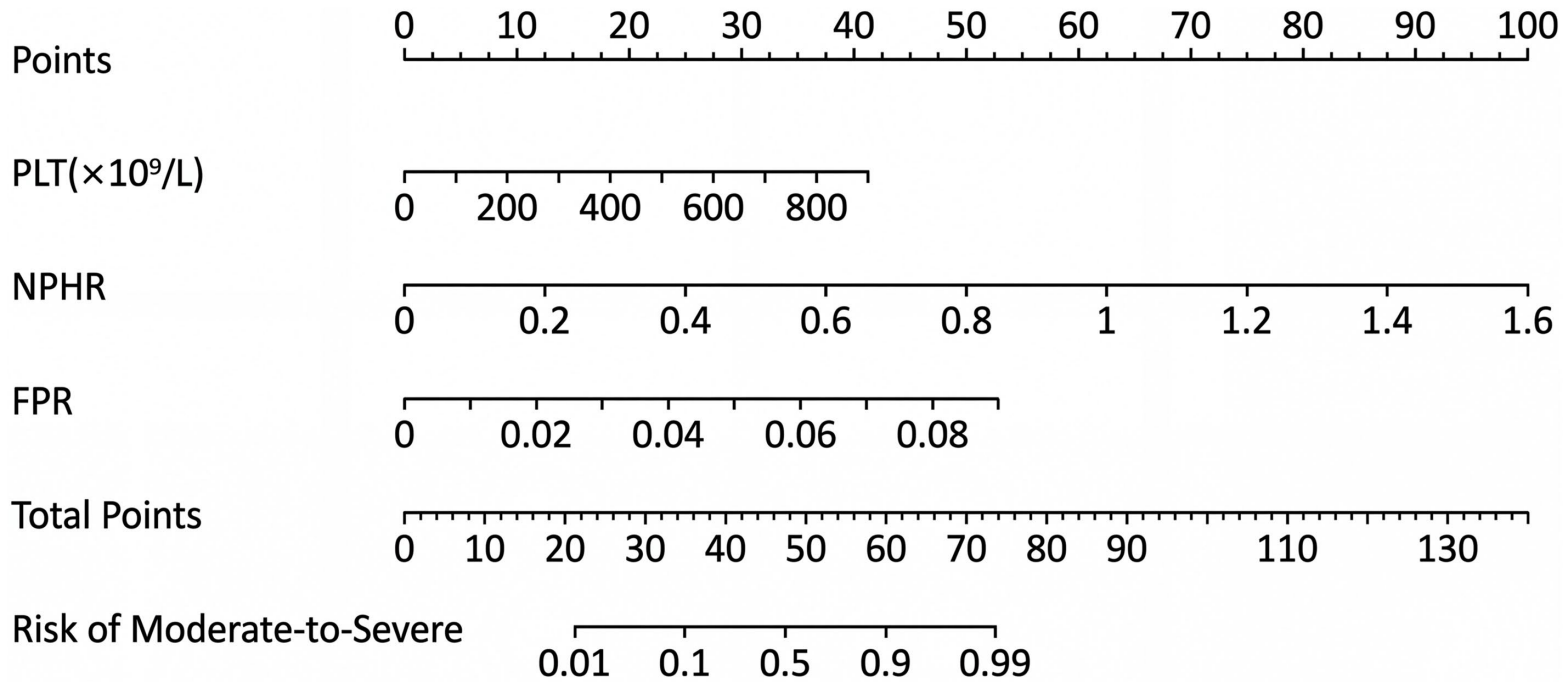
Nomogram for predicting moderate-to-severe UC. PLT, platelet count; NPHR, neutrophil percentage-to-hemoglobin ratio; FPR, fibrinogen-to-prealbumin ratio.

**Figure 3:**
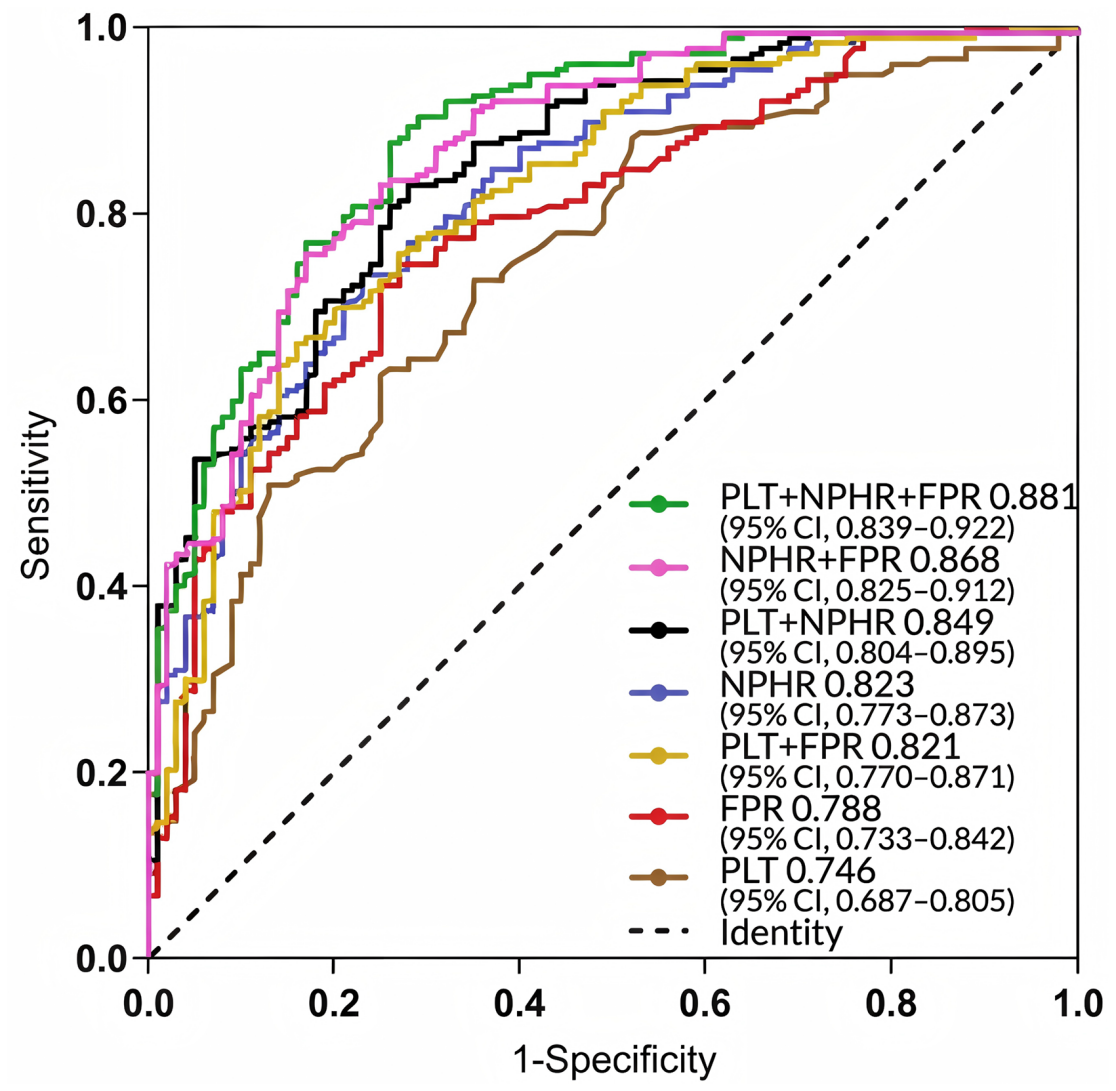
ROC curves for PLT, NPHR, and FPR in the evaluation of moderate-to-severe UC. PLT, platelet count; NPHR, neutrophil percentage-to-hemoglobin ratio; FPR, fibrinogen-to-prealbumin ratio.

**Figure 4:**
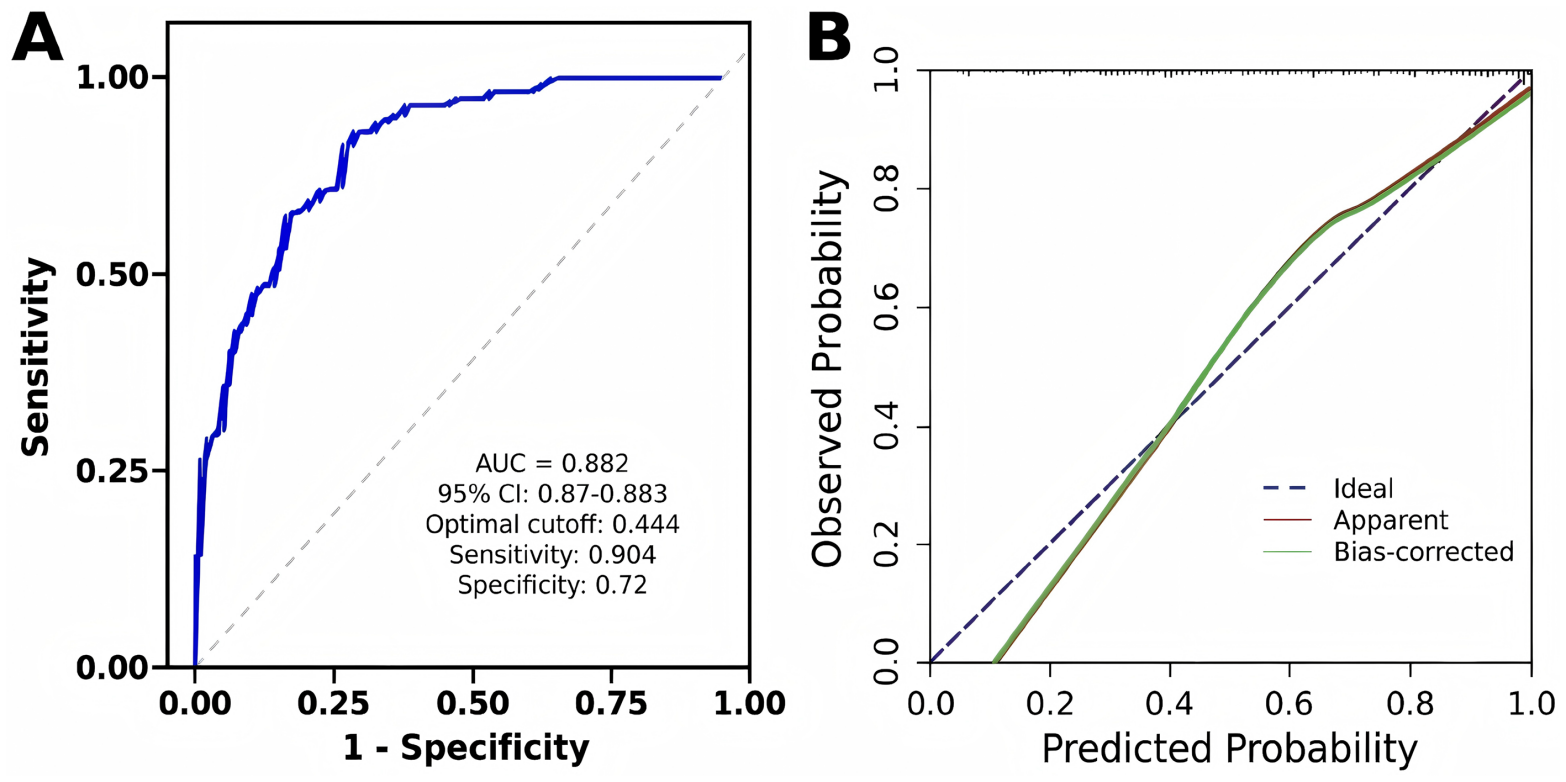
Internal validation and calibration curves of the model for predicting moderate-to-severe UC. **(A)** Bootstrap curve for predicting moderate-to-severe UC. **(B)** Calibration curve for predicting moderate-to-severe UC.

A prediction model incorporating PLT, NPHR, and FPR was developed, and a nomogram was generated. The model equation was as follows: Logit *p* = 0.008 * PLT + 10.978 * NPHR + 103.158 * FPR − 8.324 (Figure 2). ROC curve analysis for the statistically significant variables (PLT, NPHR, and FPR) showed that among the individual markers, NPHR had the best predictive performance, with an AUC of 0.823 (95% CI: 0.773–0.873), an optimal cutoff value of 0.462, a sensitivity of 73.4%, and a specificity of 77.0%. The AUC of PLT was 0.746 (95% CI, 0.687–0.805), with an optimal cutoff value of 235.500, a sensitivity of 72.9%, and a specificity of 65.0%. The AUC of FPR was 0.788 (95% CI, 0.733–0.842), with an optimal cutoff value of 0.014, a sensitivity of 74.6%, and a specificity of 73.0%. The combined model of these three markers showed the best predictive performance, with an AUC of 0.881 (95% CI, 0.839–0.922), an optimal cutoff value of 0.474, a sensitivity of 87.6%, and a specificity of 74.0% (Table 6 and Figure 3). Internal validation using 1,000 bootstrap resamples resulted in a bias-corrected C-index of 0.882 (95% CI, 0.870–0.883), yielding sensitivity of 90.4% and specificity of 72.0%, indicating good discrimination (Figure 4A). The Hosmer–Lemeshow test showed *χ*^2^ = 12.851 and *p* = 0.117, suggesting acceptable agreement between predicted and observed risks (Figure 4B).

**Table 6:** Predictive value of PLT, NPHR, and FPR for moderate-to-severe UC.

Variables	AUC	95% CI	*p*	Cut-off	Sensitivity (%)	Specificity (%)
PLT	0.746	0.687–0.805	<0.001	235.500	72.9	65.0
NPHR	0.823	0.773–0.873	<0.001	0.462	73.4	77.0
FPR	0.788	0.733–0.842	<0.001	0.014	74.6	73.0
PLT + NPHR	0.849	0.804–0.895	<0.001	0.524	83.1	72.0
PLT + FPR	0.821	0.770–0.871	<0.001	0.648	66.1	84.0
NPHR + FPR	0.868	0.825–0.912	<0.001	0.623	75.7	83.0
PLT + NPHR + FPR	0.881	0.839–0.922	<0.001	0.474	87.6	74.0

PLT, platelet count; NPHR, neutrophil percentage-to-hemoglobin ratio; FPR, fibrinogen-to-prealbumin ratio.

### 3.5 Decision curve and clinical impact curve analysis

**Figure 5:**
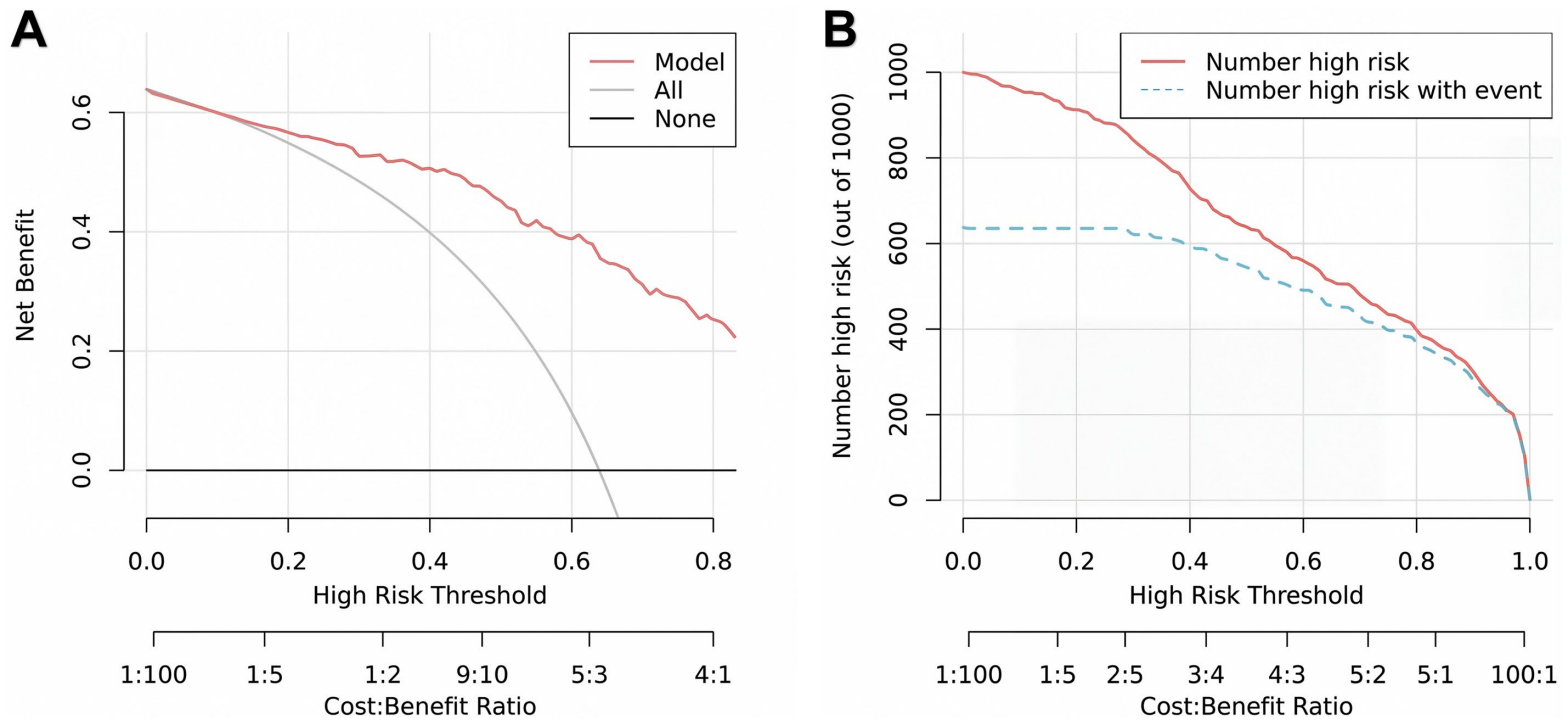
Efficacy of the model for predicting moderate-to-severe UC. **(A)** Decision curve analysis for predicting moderate-to-severe UC. **(B)** Clinical impact curve for predicting moderate-to-severe UC.

Decision curve analysis (DCA) revealed that across a wide range of threshold probabilities (approximately 0.16–1.00), the use of this prediction model provided higher net benefit, supporting its clinical utility (Figure 5A). The CIC further indicated good concordance between the model-predicted high-risk population and the observed number of events within threshold probabilities of 0.30–0.90 (Figure 5B), supporting the clinical utility of the nomogram-based risk stratification.

## 4 Discussion

NEUs are the most abundant subset of circulating leukocytes and serve as key effectors in the innate immune response (14). They rapidly migrate to sites of pathogen invasion, playing a central role in early host defense. However, excessive accumulation and persistent activation of NEUs within the intestinal mucosa can worsen epithelial barrier disruption and mucosal ulceration through the release of various effector molecules, including reactive oxygen species, proteases, and neutrophil extracellular traps, thereby exacerbating disease progression (15). In UC patients, particularly during active phases, the extent of colonic neutrophil infiltration correlates closely with disease activity and can predict adverse outcomes, such as the need for corticosteroid therapy, hospitalization, and surgical intervention (16, 17). Increased neutrophil infiltration is associated with higher disease activity and greater disease severity (18). Notably, neutrophil elastase activity is significantly elevated in UC patients, and the resulting mucosal barrier damage has been identified as a key pathological mechanism driving disease onset and progression (19). In line with these findings, the present study observed that patients with moderate-to-severe UC had a significantly higher NEU% and markedly lower Hb levels compared to those with non-moderate-to-severe disease. Anemia is one of the most common extraintestinal manifestations of UC, impairing patients’ health-related quality of life and being closely linked to cognitive dysfunction (20). The reduction in Hb levels in UC patients is multifactorial, driven by recurrent intestinal bleeding, chronic inflammation, disturbed iron metabolism, and suppression of erythropoiesis. Under inflammatory conditions, cytokines such as interleukin-6 (IL-6) upregulate hepcidin expression, promoting ferroportin degradation and thereby restricting both intestinal iron absorption and the release of stored iron (21). This mechanism underlies anemia of inflammation. Additionally, inadequate nutritional intake can exacerbate the decline in Hb levels, synergizing with these pathological processes to further promote the development and progression of anemia.

Based on the mechanisms described, NPHR is not merely a statistical composite index but a composite phenotype that reflects the dual pathological processes of neutrophil-driven inflammation and inflammation-related anemia. In recent years, composite hematological indices that integrate leukocyte counts and hemoglobin levels have gained prominence for assessing systemic inflammation and anemia in malignancies. Zhao et al. (22) first introduced the concept of NPHR, proposing that it captures the systemic imbalance between inflammatory and hematological status. Their study demonstrated significantly elevated NPHR levels among patients with non-small cell lung cancer, highlighting its potential in distinguishing benign from malignant pulmonary nodules. Additionally, NPHR has been closely linked to poor prognosis in cardiovascular diseases, such as severe chronic heart failure and atrial fibrillation (23, 24). Given the pronounced inflammatory responses and anemia observed in UC patients, the present study explored NPHR’s potential to predict UC disease activity and severity. Our results revealed a significant correlation between NPHR and all four UC activity scores. Notably, NPHR showed a correlation coefficient of 0.693 with the modified Mayo score and an AUC of 0.823 for predicting moderate-to-severe UC, demonstrating strong predictive value. In UC, an elevated NPHR reflects heightened innate immune activation coupled with anemia characterized by iron-restricted erythropoiesis, thereby capturing the overall pathophysiological burden of the disease more comprehensively than any single inflammatory marker.

FIB is not only a key substrate in the coagulation cascade but also a critical mediator linking coagulation, inflammation, and immune regulation (25–27). As an acute-phase reactant, FIB levels rise in various inflammatory and stress conditions, correlating with the severity and prognosis of multisystem diseases, including liver disease, cardiovascular disease, and certain neurological disorders (28–32). FIB promotes leukocyte transendothelial migration and regulates the adhesion, activation, and cytokine release of inflammatory cells through interactions with receptors such as integrins, suggesting that FIB not only reflects a hypercoagulable state but also actively participates in inflammatory amplification (33). In UC patients, where chronic inflammation and hypercoagulability coexist, elevated FIB levels partially reflect the acute-phase response following mucosal damage and the procoagulant microenvironment. Furthermore, FIB levels correlate with disease activity, indicating its usefulness as a convenient biomarker for UC assessment (7, 34, 35). Patients with inflammatory bowel disease (IBD) are at high risk of malnutrition, a condition that becomes more pronounced during active UC. PA, a negative acute-phase protein with a short half-life (36), more effectively reflects short-term nutritional changes than albumin. Its levels are influenced by factors such as inflammation, infection, stress, and hepatic function (37). A decrease in PA not only indicates impaired nutritional status but also reflects inflammatory activation. During active UC, the massive release of pro-inflammatory cytokines, such as tumor necrosis factor-α and IL-6, from the intestinal mucosa induces an acute-phase response that suppresses albumin and PA synthesis in hepatocytes, reducing PA production (38). Additionally, persistent intestinal inflammation disrupts the mucosal barrier, increasing permeability and leading to protein leakage and impaired amino acid absorption (39). These processes exacerbate the deficiency of substrates necessary for PA synthesis, contributing to reduced PA levels, which closely mirror disease activity in UC patients (40). Low PA levels, especially values ≤15 mg/dL, are associated with prolonged hospital stays, higher short-term readmission risk, and elevated inflammatory markers and endoscopic activity (9), suggesting that PA is a valuable indicator of inflammatory burden, nutritional status, and clinical outcomes in UC.

The FPR integrates coagulation function and nutritional status, providing a comprehensive measure. An elevated FPR indicates a severe disease stage characterized by high inflammatory burden, hypercoagulability, and significant nutritional depletion—conditions typically associated with more severe endoscopic findings and a more aggressive clinical course. While the prognostic value of FPR in gastrointestinal malignancies has been well established (41), research in UC remains limited. Available data from IBD subgroups suggest that FPR outperforms CRP and ESR in assessing UC disease activity, highlighting its significant clinical potential (42). In the present study, FPR was significantly elevated in patients with moderate-to-severe disease and showed strong correlations with all four disease activity scores, further validating its applicability in the UC population (43).

As anucleate cell fragments rich in active granules (44), PLTs are essential not only for hemostasis but also for immunothrombosis. Through pattern recognition receptor signaling, interactions with endothelial cells, and crosstalk with immune cells, PLTs release chemotactic and inflammatory mediators, influencing inflammatory regulation and tissue injury (45). In autoimmune disorders, including systemic lupus erythematosus and rheumatoid arthritis, platelet activation is strongly linked to thrombosis and organ damage (46, 47), making PLTs a validated marker for disease activity (48). Similarly, the potential role of PLTs in UC merits further exploration. Reactive thrombocytosis is commonly observed in active UC, with PLTs correlating with disease activity and inflammatory markers, suggesting their potential as auxiliary indicators for disease assessment (8, 49, 50). Our study further confirmed that PLTs were notably increased in patients with moderate-to-severe disease and represented an independent predictor of disease severity.

ROC analysis revealed an AUC of 0.746, indicating that PLTs have moderate discriminative ability for identifying moderate-to-severe disease activity. This elevation is likely driven by inflammation-induced thrombopoiesis and inflammation-coagulation coupling. Chronic inflammatory cytokines like IL-6 promote megakaryocyte differentiation and platelet production. Under procoagulant conditions, the increased risk of microthrombi formation may further disturb local microcirculatory perfusion (51, 52). Activated platelets also release molecules like CD40L, amplifying the inflammatory response through interactions with leukocytes (53). Thus, PLTs in UC reflect both systemic inflammation and coagulation system activation. Persistent elevation suggests significant mucosal involvement and poor prognosis, highlighting the need for validation of PLT’s role in disease stratification through future prospective studies.

From a pathophysiological perspective, NPHR, FPR, and PLT assess distinct yet interconnected axes of inflammation, nutritional status, and coagulation in active UC. NPHR captures both innate immune activation and acute inflammatory burden (via elevated NEU%). By incorporating Hb levels, it integrates chronic factors related to mucosal inflammation, including blood loss, inflammation-driven iron dysregulation, and inflammatory anemia. Therefore, NPHR offers a more comprehensive assessment of disease activity and systemic depletion than individual inflammatory markers. FPR serves as a dual marker: elevated FIB reflects the acute-phase response and hypercoagulability, while reduced PA signals nutritional impairment and suppressed synthesis. This effectively reveals a vicious cycle of intensified inflammation, deteriorating nutrition, and heightened hypercoagulability. PLT elevation typically represents reactive thrombocytosis induced by inflammatory stimuli. Beyond hemostasis, activated PLTs release pro-inflammatory mediators and interact with neutrophils and fibrinogen, promoting microcirculatory hypercoagulability and mucosal injury, which further exacerbates inflammation. In conclusion, these markers provide complementary insights into inflammatory burden, somatic depletion, and the inflammation-coagulation crosstalk. This mechanistic rationale supports their identification as independent risk factors and highlights the superior predictive value of the combined model.

Although the findings of this study are encouraging, several limitations of the present study should be noted. First, it was a retrospective, single-center study with a relatively small sample size, and the moderate-to-severe group was markedly larger than the low-activity group, which may have introduced selection bias and reduced the generalizability of the results. Moreover, because of the study’s observational design, only links between variables can be demonstrated, precluding the establishment of definitive cause-and-effect relationships. In addition, retrospective data collection may be subject to information bias, unmeasured confounding, and missing variables. Second, the absence of an external validation cohort restricts the robustness and broader applicability of the results. Third, endoscopic evaluations were conducted by different physicians, potentially introducing inter-observer variability. Future studies should establish standardized protocols or employ central reading to minimize this issue. Lastly, this study did not include a direct comparison with existing non-invasive reference biomarkers, such as FC and CRP. Among them, FC was not included in the analysis due to some missing data. Therefore, future large-scale, multicenter, and prospective studies incorporating independent external validation cohorts and direct comparisons with established biomarkers are warranted to further clarify the clinical utility of NPHR, FPR, and PLT in the assessment of UC.

## 5 Conclusion

In conclusion, serum NPHR, FPR, and PLT are non-invasive, cost-effective, and easily accessible biomarkers that effectively quantify inflammatory burden and disease staging. These indicators demonstrate significant clinical value and can serve as crucial tools in the management of UC. They facilitate timely clinical interventions and the formulation of personalized treatment strategies, thereby improving patient prognosis. Future research is warranted to elucidate the dynamic evolutionary patterns of these inflammatory indicators in relation to UC activity and severity.

## Data Availability

The raw data supporting the conclusions of this article will be made available by the authors, without undue reservation.
